# Digital Cognitive Behavior Therapy Intervention for Depression and Anxiety: Retrospective Study

**DOI:** 10.2196/21304

**Published:** 2020-08-26

**Authors:** Aarathi Venkatesan, Lily Rahimi, Manpreet Kaur, Christopher Mosunic

**Affiliations:** 1 Vida Health San Francisco, CA United States

**Keywords:** depression, anxiety, CBT, digital mental health intervention, digital therapeutics, cognitive behavioral therapy, therapy, digital health, intervention, mental health

## Abstract

**Background:**

Digital mental health interventions offer a scalable solution that reduces barriers to seeking care for clinical depression and anxiety.

**Objective:**

We aimed to examine the effectiveness of a 12-week therapist supported, app-based cognitive behavioral therapy program in improving symptoms of depression and anxiety within 9 months.

**Methods:**

A total of 323 participants with mild to moderately severe depression or anxiety were enrolled in a 12-week digital cognitive behavior therapy program. The analysis was restricted to participants who provided at least one follow-up assessment after baseline. As a result, 146 participants (45.2%) were included in the analysis—74 (50.7%) participants completed assessments at 3 months, 31 participants (21.2%) completed assessments at 6 months, and 21 participants (14.4%) completed assessments at 9 months. The program included structured lessons and tools (ie, exercises and practices) as well as one-on-one weekly video counseling sessions with a licensed therapist for 12 weeks and monthly check-in sessions for 1 year. The clinically validated Patient Health Questionnaire (PHQ-8) and Generalized Anxiety Disorder Scale (GAD-7) were used to assess depression and anxiety, respectively. Linear mixed-effects modeling was employed to examine changes in depression and anxiety over time.

**Results:**

We observed a significant positive effect of program time on improvement in depression (β=–0.12, *P*<.001) and anxiety scores (β=–0.10, *P*<.001). At the end of the 12-week intervention, we observed an average reduction of 3.76 points (95% CI –4.76 to –2.76) in PHQ-8 scores. Further reductions in depression were seen at program month 6 (4.75-point reduction, 95% CI –6.61 to –2.88) and program month 9 (6.42-point reduction, 95% CI –8.66 to –6.55, *P*<.001). A similar pattern of improvement emerged for anxiety, with a 3.17-point reduction at the end of the 12-week intervention (95% CI –4.21 to –2.13). These improvements were maintained at program month 6 (4.87-point reduction, 95% CI –6.85 to –2.87) and program month 9 (5.19-point, 95% –6.85 to 4.81). In addition, greater program engagement during the first 12 weeks predicted a greater reduction in depression (β=–0.29, *P*<.001)

**Conclusions:**

The results suggest that digital interventions can support sustained and clinically meaningful improvements in depression and anxiety. Furthermore, it appears that strong initial digital mental health intervention engagement may facilitate this effect. However, the study was limited by postintervention participant attrition as well as the retrospective observational study design.

## Introduction

Telemedicine delivered through smartphone-based apps is fast redefining how care is sought and delivered for disease prevention and management. The emergence of digital health technology is particularly relevant for mental health services. With a 12-month prevalence rate of 6.7% and 3.1% for major depressive disorder and generalized anxiety disorder, respectively, in the United States [1], depression and anxiety are the leading causes of disability and lost productivity [2]. Nevertheless, there are barriers unique to mental health that hinder timely access to care. Besides affordability and accessibility issues, stigma surrounding mental illnesses [3], lack of motivation, and low rates of mental health literacy [4] significantly impact willingness to seek treatment and contribute to low engagement with mental health services.

With the rapid development and application of digital technologies such as smartphone apps, telemedicine has emerged as a compelling solution for disease treatment and management. Digital mental health interventions are growing in appeal because they offer scalable solutions and can improve uptake rates among those who are less inclined to seek mental health services. Digital mental health interventions are an effective means of delivering evidence-based psychological treatment to patients who are unable or unlikely to seek in-person treatment with a provider [5]. In addition, digital mental health interventions also appear to improve access, reduce stigma, and ease pressure when compared to face-to-face services [6].

Given the potential benefits of digital mental health interventions, there has been increased research focusing on their effectiveness. In their meta-analytic review, Richards et al [7] observed a significant and clinically meaningful effect of internet-based interventions for the treatment of generalized anxiety disorder when compared to waitlist controls, including those with comorbid depression. Similarly, in a meta-analysis of randomized controlled trials that employed smartphone-based mental health interventions, Firth et al [8] observed a significant moderate treatment effect of internet-based interventions on depressive symptoms when compared to both waitlist and active controls; a similar treatment effect was observed for anxiety [9].

While early evidence is promising, digital mental health interventions are not monolithic in their treatment approach, design, or delivery. They can be game-based, focus primarily on psychoeducation, or incorporate human interactions. Digital mental health interventions can entail one-on-one interactions in real time with a therapist via audio or video conferencing tools and involve both synchronous and asynchronous text messaging.

Interestingly, Firth et al [8] observed that smartphone-only apps were more effective than interventions that included a human or computerized interaction were and hypothesized that smartphone-only apps may be more comprehensive and self-contained in content and experience than interventions that relied on external input are. Li et al [10] observed a similar pattern: a larger effect size for game-based interventions for the treatment of depression that did not include therapist support than for therapist-supported interventions. On the other hand, Spek et al [11] noted the opposite pattern, wherein a stronger effect size was observed for therapist-supported internet-based cognitive behavior therapy interventions for anxiety symptoms. It is important to note here that digital mental health interventions can vary substantially in their therapeutic approach from a focus on mindfulness and psychoeducation to therapist-supported digital cognitive behavior therapy. Indeed, Firth et al [8] did observe cognitive behavior–based interventions were associated with significantly greater reductions in depression compared to those associated with other approaches. Additionally, research suggests that internet-based cognitive behavior therapy programs are roughly equivalent in effectiveness compared to those of face-to-face therapy [12,13].

Digital mental health interventions are not without their drawbacks. Importantly, it is still unclear if all digital psychological treatments are equally effective. For instance, in their review of digital mental health interventions for students, Lattie et al [14] found that web-based interventions were effective in improving depression, anxiety, and psychological well-being. However, they noted participant attrition and program discontinuation across studies, suggesting that sustaining patient engagement remains a continued challenge for digital interventions.

In summary, recent interest and demand for remotely delivered mental health interventions has led to an increase in app-based solutions. Technology-based mental health programs have been shown to be effective in improving access and treating common mental health conditions such as depression. However, given the heterogeneity of digital mental health interventions in terms of treatment approach and modality and continued challenges of attrition, their overall effectiveness remains an open question. Though a fairly robust body of academic research supports the efficacy of digital mental health interventions in the treatment of mild to moderate depression and anxiety, there remain gaps. Specifically, commercially available, app-based mental health programs have received less research scrutiny, particularly, in evaluating medium-term and long-term outcomes. Second, hybrid models that combine a cognitive behavior therapy–based digital intervention with one-on-one remote therapist support have been assessed to a lesser extent.

In this study, we evaluated the Vida Health therapy program, a digital therapeutic intervention for mild to moderate depression and anxiety. Vida Health is a commercially available, app-based digital health program for mental health and cardiometabolic conditions. The program is available direct to consumers, as an employee-based benefit or via select health plans. The Vida Health app offers tailored digital content paired with remote therapy and health coaching with licensed therapists, registered dieticians, certified diabetes educators or health education specialists. Vida Health program offerings include cognitive behavior therapy, mindfulness-based stress management, weight management, and chronic disease (eg, type 2 diabetes) management. Available in all 50 states, the therapy program that is the focus of this study is based on cognitive behavior therapy, a therapeutic approach that has shown the strongest evidence for treating depression and anxiety [15,16]. Cognitive behavior therapy stands out as the leading therapeutic modality for depression and anxiety, as it is both shorter in duration, and its positive outcomes last longer, as compared to those of other therapeutic modalities [17]. Our primary objective was to assess the long-term outcomes from a digital mental health intervention incorporating evidence-based psychotherapeutic content along with one-on-one sessions with a remote licensed therapist. Our secondary objective was to examine if intervention engagement was associated with stronger outcomes at the end of the intervention. This study also examined depression and anxiety improvements during the postintervention maintenance phase.

## Methods

### Design

A retrospective observational study design was employed to evaluate changes in depression and anxiety following Vida Health’s app-based cognitive behavior therapy program that included one-on-one therapist-supported counseling. The study was approved by an independent institutional review board (Western institutional review board). The institutional review board waived the informed consent because the study was identified as having minimal risk and the data were fully anonymized before retrospective analysis.

### Measures

Depression was assessed using the clinically validated 8-item Patient Health Questionnaire (PHQ-8). Although the PHQ-9 may be more widely used, we utilized the PHQ-8 in this program. The key distinction between the two instruments is the inclusion of the suicidality item in the PHQ-9. In lieu of screening via app, all participants were evaluated for suicidality by a licensed therapist during the initial biopsychosocial assessment and intake. The 7-item Generalized Anxiety Disorder (GAD-7) scale was used to evaluate anxiety. These validated self-reported instruments are commonly used in clinical practice to assess depression and generalized anxiety disorder [18,19]. The PHQ-8 and GAD-7 assessments were automatically administered in the app every 2 weeks for the duration of the study. Although participants were encouraged to complete the assessments on the day of receipt, they had the opportunity to complete the assessments at any time during the 2-week period, after which, the next scheduled assessment became available. Additional assessments could also be sent at any time by the therapist, at their discretion. The semimonthly assessment schedule was selected in order to provide therapists with periodic insight into a participant’s progress in the program and assess the possible need for additional care or referral to external resources.

### Study Sample and Recruitment

**Figure 1:**
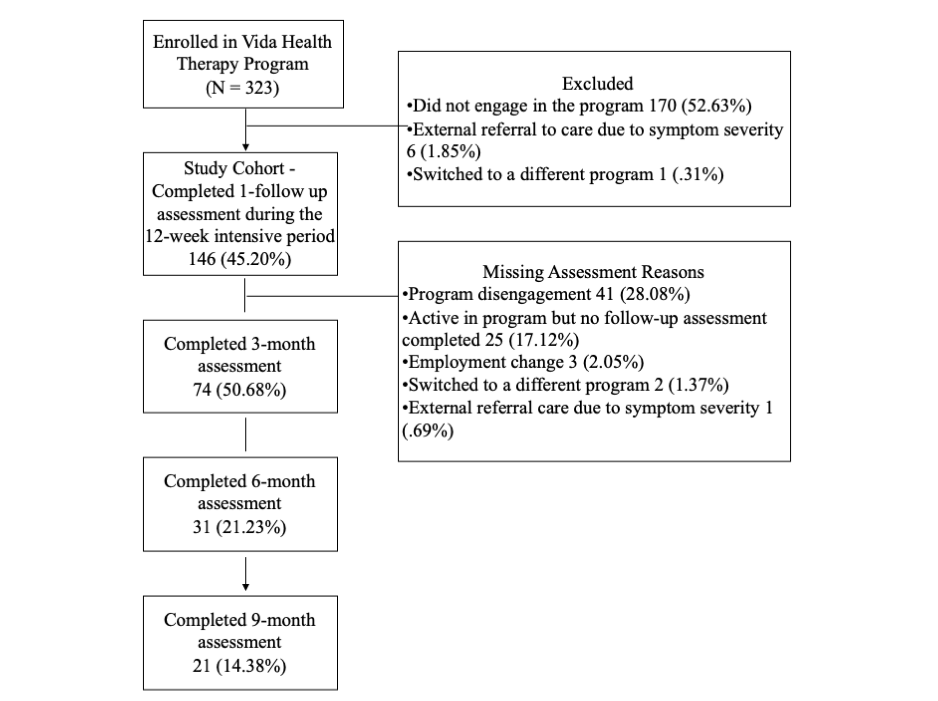
Schematic of participant flow.

The study included adults (18 years or older) who owned a smartphone or tablet, were fluent in spoken and written English, with mild to moderately severe depression or mild to moderate anxiety. Participants were recruited from companies based in the California Bay Area and in the state of Washington that offered the Vida Health cognitive behavior therapy program as a benefit to employees and spouses. Although Vida Health therapy program patients are currently distributed across the United States, this study focused on participants drawn from organizations where participants had been enrolled in the program for at least 6 months. Between February 2017 and January 2020, 323 participants were recruited through a combination of email announcements, paper flyers, and onsite events at their employers (Figure 1). Participants were directed to download the Vida Health app from the Apple App Store (iOS version) or Google Play Store (Android version) and enroll using an invite code to confirm eligibility.

Prior to enrolling in the Vida Health digital cognitive behavior therapy program, participants completed a brief set of registration questions that included name, contact information, basic demographics (age, gender, height, and weight), and existing health conditions. At program start, participants completed the Patient Health Questionnaire (PHQ-8) and Generalized Anxiety Disorder scale (GAD-7). Study enrollment and participant flow are also presented in Figure 1. Participants with PHQ-8 scores between 5 and 19 or GAD-7 scores between 5 and 14 were included in the study. Participants who scored in severe depression (PHQ-8 score>19) or severe anxiety (GAD-7 score>14) ranges were ineligible for the program. Furthermore, if during the initial intake or any time during the intervention a participant presented with or reported any current or recent active symptoms or conditions that would likely be better supported by an alternative source of care, the therapist explained this to the participant and referred the participant to an alternative care resource or to a Vida Health care navigator for help getting connected to an alternative source of care. Conditions that were the basis for an individual being excluded from program participation included eating disorders, substance use disorders, suicidality, homicidality, acute posttraumatic stress disorders, episodes of mania, or psychosis. Participants with subthreshold or meeting criteria for a psychiatric diagnosis outside of mild to moderate anxiety and or depression were excluded from the study. Participants who were ineligible for the program were referred to a Vida Health care navigator and provided with a list of sources to seek care through in-person clinical services. Vida Health care navigators are licensed mental health professionals who connect ineligible participants to alternative care resources, including in-person treatment through local community counseling centers, private practice providers, employee assistance programs, or referral to an in-network care provider through the participant’s health insurance.

Study participants who were eligible were then paired with a licensed therapist based on their state of residence and their preferred times for consultations. Therapists were mental health professionals licensed by their states’ respective licensure boards and employed by Vida Health. Therapists were licensed in one or more states. License titles and types, and scope of license-practicing privileges varied by state. Therapists had one or more of the following license types (the list below is not all inclusive): licensed clinical social workers, licensed medical clinical social workers, licensed independent social worker, licensed marriage and family therapists, licensed professional counselors, licensed professional clinical counselors, licensed mental health counselor, licensed clinical psychologists, licensed practicing psychologist, and clinical mental health counselor.

Prior to program intake, all participants received and signed an informed consent to psychotherapy form. Consent to psychotherapy is a standard of care practice at the start of therapy. Its purpose is to inform clients of the expectations of therapy, their rights to confidentiality, and limits to confidentiality (including mandated reporting requirements as stipulated by the therapist’s licensing board and state regulations) and supply provider license information. The consent form also included details about the termination process, accessibility through digital text messaging, and video conferencing; the cancellation policy; limitations to teletherapy; and consent to engage in teletherapy. During the first appointment, the therapist conducted a biopsychosocial intake questionnaire that reviewed history of previous treatment or diagnosis, family history of mental health conditions and physical health conditions, current support systems, current medical conditions, current prescribed medications, history of substance use, strengths, current interests, spirituality or religiousness, sleep quality and quantity, appetite, current presenting problem, presenting symptoms, and mental status examination (including assessment of mood, affect, orientation, attire, eye contact, thought process, attention, auditory or visual hallucinations, delusions, and any presenting suicidal or homicidal ideation). After completing the intake, the therapist generated an initial diagnostic impression and determined if a participant was eligible to continue participating in the program. If a participant presented with mild to moderate anxiety or depression, they were eligible to continue the program.

### Therapeutic Approach and Intervention

The Vida Health cognitive behavior therapy program is a HIPAA-compliant structured digital program that connects adults living with mild to moderate depression or anxiety with a licensed therapist. Although the core structure, duration, and focus of the digital cognitive behavior therapy program remained the same, certain app-features, improved functionality, and app-enhancements were introduced to the cognitive behavior therapy program between 2017 and 2020, resulting in 3 different program iterations. These program versions were included as a random factor in the final linear mixed methods model, to account for any potential differences in version effectiveness.

**Figure 2:**
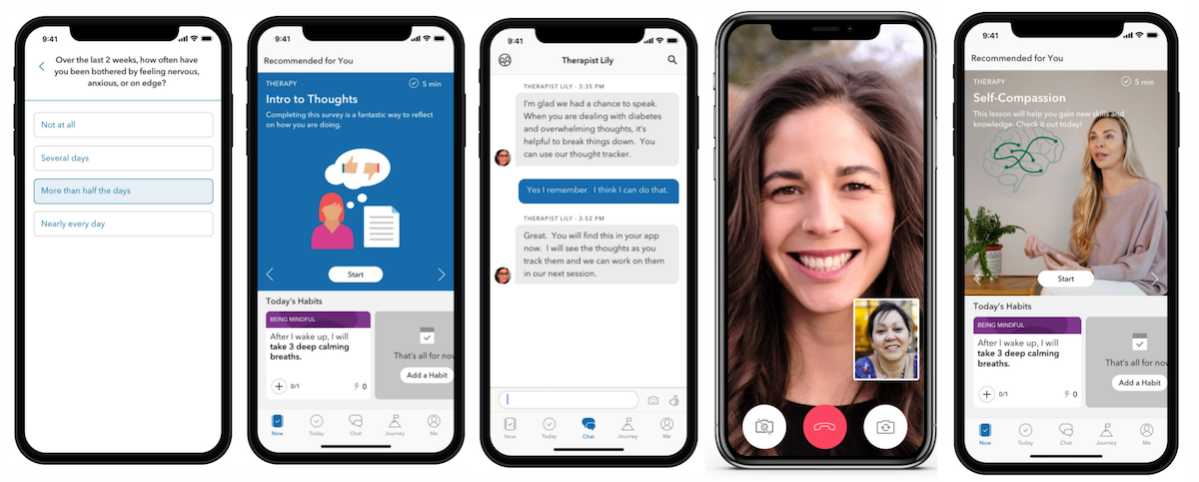
Vida Health Therapy program screens.

As part of the intervention, participants were sent audio-, video-, and text-format lessons, activities and practices based on cognitive behavior therapy by their therapist through the app. Cognitive behavior therapy addresses maladaptive thinking patterns by exploring the relationship between thoughts, emotions, and behavior [20]. The materials sent to the participants through the Vida Health app reviewed core concepts of cognitive behavior therapy including the cognitive model, guided discovery, identifying cognitive distortions, behavioral activation, and techniques for addressing maladaptive thinking [20,21]. Lessons and activities included questions in an effort to support increased awareness of underlying thinking patterns and to facilitate the practice of alternative, more adaptive thoughts. Participants could select from multiple choice options, checklists, and free text, as well as review concepts through reading, listening to audio practices, or watching videos (see Figure 2).

The therapist reviewed the completed lessons prior to every weekly consult, and in discussion with the participant, reviewed strategies for applying the concepts and skills that had been covered. The therapist would check-in with the participant about their current mood, set goals for the week, and prepare them for the upcoming lessons and activities as homework for the week ahead. Over the course of the program, the therapist met with the participants on a weekly basis for a duration of 30 minutes over video or phone call. Every week, the therapist generated a personalized treatment plan that reviewed the participant’s short-term and long-term goals as well as the habits, actions, and homework they planned to engage in for the week. These treatment plans were reviewed with the participant during the core intervention phase. Toward the end of the intensive phase (at approximately week 10), the therapist sent the participant lessons and tools to help generate a Wellness Recovery Action Plan. The Wellness Recovery Action Plan is a plan that identifies known triggers and coping strategies that the participant has identified to support their management of symptoms [22]. The Wellness Recovery Action Plan is designed to support both maintenance of improved symptoms and functioning as well as prevent relapse.

### Statistical Analyses

Across all participants, the mean survey completions were 5.75 (SD 3.90) and 5.50 (SD 3.50) for GAD-7 and PHQ-8, respectively. The study design intended to obtain at least one assessment every fortnight. Based on this expectation, we could have received as many 1106 completed PHQ-8 surveys, assuming one survey completion per participant per fortnight; we received 698 PHQ-8 assessments, indicating a survey completion rate of 63.11%. Likewise, out of the 1185 possible GAD-7 survey completions (one per participant per fortnight), we received a total of 744 surveys indicating a completion rate of 62.78%. The temporal resolution of changes in PHQ-8 or GAD-7 scores was far higher for some participants than others. Although the automated assessments were administered every 2 weeks (with additional ones administered by the therapist), a participant could complete the survey at any time during the 2-week period. As a result, a participant could have completed more than one survey within a fortnight. For example, a participant could complete a survey received on week 12, on the day of receipt or 8 days thereafter. The week of survey completion was calculated as the difference in weeks from program start. As a result, on any given program week, only a fraction of participants contributed data, with no consistent cadence. To overcome this data sparsity, curve fitting was applied as a data imputation technique for each participant. All data preparation was performed using the curve_fit function (SciPy library, version 1.5.2 Python, version 3.7.7) [23]. The objective of the analysis was to evaluate changes (from baseline) in depression and anxiety over time for all participants. Data imputation through curve fitting was employed in order to provide participants with equal representation and weighting for all the days from program start to the last program day on which data was available for a participant. Data extrapolation beyond the last day on which a participant had a valid PHQ-8 was not performed due to potential artifacts. As a result, participants did not contribute to the analyses beyond the last day for which they had data. When data was aggregated by program month, participants whose last survey completion occurred during the month, contributed correspondingly fewer points to the estimate. The study cohort included participants who had only one baseline assessment and no follow-up. To assess any baseline differences between program non-starters and program completers, a two-tailed chi-squared analysis was performed.

Care was taken to ensure fidelity of data due to known issues with automated digital data acquisition platforms. Specifically, during onboarding, duplicate entries that could have occurred by participants inadvertently submitting the survey twice were ignored. Furthermore, partially completed assessments were not included in the analysis. For each participant, the timeseries of available PHQ-8 scores was compiled after removing duplicate entries. Three different functional forms were fit to this data: linear, quadratic, and sigmoidal (generalized inverse), and the fit that yielded the lowest root mean squared error was chosen as each participant’s trajectory. A nonlinear fit was applied only in instances where 6 or more data points were available. Furthermore, for the sigmoidal fit, the magnitude of the coefficients was capped. These two steps were necessary to prevent overfitting. In order to preserve temporal resolution inherent in the data, during curve fitting, predicted scores were calculated for each program day. Predicted scores were then aggregated by program week or month, as applicable, with a month being defined as a 4-week period. The same method was applied for GAD-7 scores.

To determine the effect of program time on changes in depression and anxiety scores, conventional methods such as ordinary least squares can be employed. However, these methods do not account for heterogeneity in the data, specifically, possible differences in the effectiveness across provider and program versions. In this study, participants were drawn from four companies, nested within three cognitive behavior therapy program versions that were nested across 30 providers. Hence, we employed a linear mixed-effects model where company, cognitive behavior therapy program version, and provider were treated as random effects. All analyses were performed using StatsModels (Python) [24]. *P* values<.05 were deemed significant.

### Data Availability

The data sets analyzed for this study are available from the corresponding author upon request.

## Results

The study included 323 participants enrolled in a digital cognitive behavioral intervention between February 2017 and January 2020 with baseline PHQ-8 scores or GAD-7 scores ≥5. The 9-month study cohort recruitment period ended in October 2019. These analyses were restricted to participants who had at least two valid survey submissions between baseline and program month 9. While the comorbidity of depression with anxiety was prevalent in the majority of participants, there were 7 participants with anxiety but subclinical levels of depression. Therefore, unless otherwise noted, results are reported by condition. Program nonstarters were defined as participants for whom no survey data was available beyond their initial baseline week. Of the 323 participants, 139 participants (43.0%) completed a follow-up PHQ-8 during the 9-month period, and 146 participants (45.2%) completed a GAD-7. There were no significant baseline differences in PHQ-8 between the treatment cohort and program nonstarters (nonstarter: mean 10.08, SD 3.78; treatment cohort: mean 10.48, SD 3.95; *P*=.26). Likewise, no baseline differences in GAD-7 scores were observed between groups (nonstarter: mean 9.19, SD 3.32; treatment cohort: mean 9.52, SD 3.70; *P*=.07). Furthermore, chi-square tests revealed no significant baseline differences between groups in the occurrence of self-reported chronic health conditions such as diabetes, obesity, hypertension, and hyperlipidemia (*P*=.59).

Demographics and baseline characteristics for depression and anxiety are shown in Table 1. While there were more women enrolled in the program than men, there were no significant gender-based differences in depression or anxiety scores during baseline (*P*=.52). For depression, 54.7% (76/139) of the cohort had baseline PHQ-8 scores that corresponded to moderate to severe depression (PHQ-8 score≥10). For anxiety, 41.8% (61/146) of the participants had GAD-7 scores≥10 corresponding to moderate to severe anxiety.

**Table 1:** Baseline characteristics by condition.

Condition and characteristic			Value	
**Depression** **(n=139)**				
	Baseline PHQ-8 score^a^, mean (SD)		10.48 (3.95)	
	Age (years), mean (SD)		36.42 (9.22)	
	**Gender, n (%)**			
		Male		42 (30.2)
		Female		95 (68.3)
		Did not specify		2 (1.4)
**Anxiety (n=146)**				
	Baseline GAD-7 score^b^, mean (SD)		9.52 (3.70)	
	Age (years), mean (SD)		36.10 (9.03)	
	**Gender, n (%)**			
		Male		41 (28.1)
		Female		104 (71.2)
		Did not specify		1 (0.7)

^a^PHQ-8: 8-item Patient Health Questionnaire.

^b^GAD-7: 7-item Generalized Anxiety Disorder.

### Depression and Anxiety Outcomes

The available PHQ-8 score assessed at the start of the program served as the intake PHQ-8 and were treated as the baseline. Change from baseline, the outcome variable, was defined as the difference between the baseline and the predicted score derived from curve-fitting to the participant’s data. Program time was included as a fixed effect as it was hypothesized that time engaged with cognitive behavior therapy program would predict reduction in depression scores. Baseline PHQ-8 and the composite engagement score were also predictors. As mentioned earlier, the trial period included 3 cognitive behavior therapy program versions. Pairwise comparisons revealed no significant baseline differences in depression or anxiety scores between groups (*P*=.58). Likewise, there were no company-based baseline differences between groups (*P*=.91). Company and cognitive behavior therapy program versions were included as the top-level group variables in the model. Providers were included in the model as a nested group and specified as a variance component.

**Figure 3:**
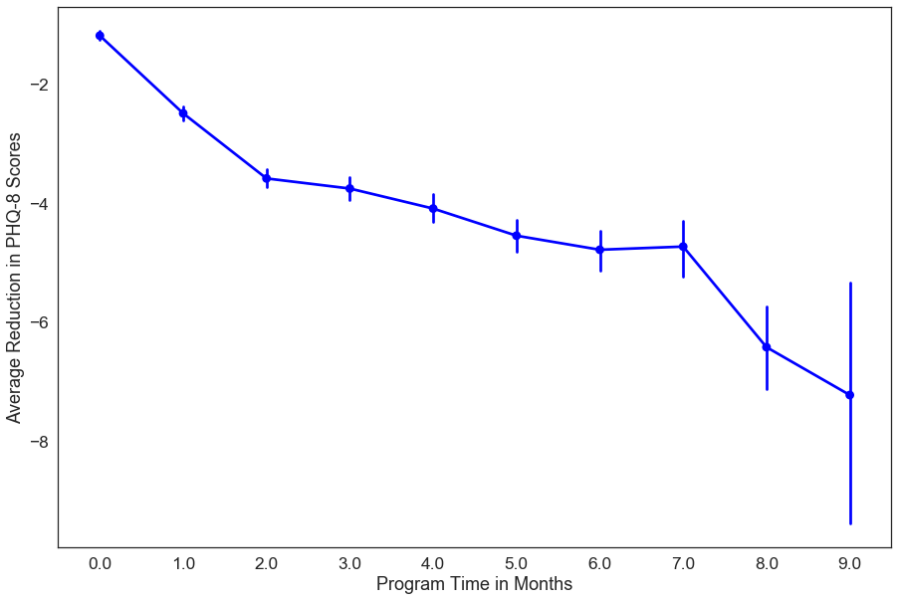
Estimated marginal mean of depression scores by cognitive behavioral therapy program time. PHQ: Patient Health Questionnaire.

Figure 3 shows the average reduction and standard error in PHQ-8 scores from baseline by program month. We observed clinically significant improvement in depression as a function of program time (β=–0.12, *P*<.001). A total of 69 PHQ-8 completions at the end of the intensive phase (69/139, 49.6% of participants at month 3), 29 completions at month 6 (29/124, 23.9% of participants at month 6), and 13 completions by month 8 (13/103, 12.6% of participants at month 9) were included in the analyses. Although 77.3% (51/66) and 58.1% (25/43) of participants remained active (ie, therapist contact, lesson completions) in the program at months 6 and 9, respectively, participants did not complete the assessments. There was an average reduction of 3.76 points (95% CI –2.76 to –4.76, *P*<.001; Hedges *g*=0.96) by the end of the intensive phase of the program (month 3). Further improvements in depression were seen during the maintenance phase of the intervention, with an average reduction of 4.75 points (95% CI –2.88 to –6.61, *P*<.001; Hedges *g*=1.10) at program month 6. By program month 9, we observed a 6.42-point average reduction (95% CI –7.15 to –5.70).

**Figure 4:**
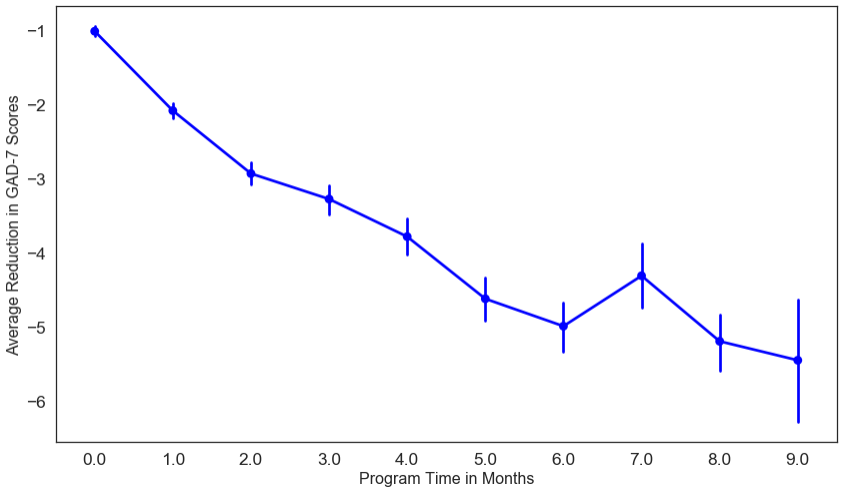
Estimated marginal mean of anxiety scores by cognitive behavioral therapy program time. GAD: Generalized Anxiety Disorder.

We observed a similar pattern of improvement in anxiety (see Figure 4). There was a significant reduction in anxiety scores with increased program time (β=–0.10, *P*<.001). A total of 50.7% of participants (74/146) completed the GAD-7 assessment at the end of the intensive phase, 23.5% of participants (31/132) who had been in the program for at least 6 months completed the GAD-7 assessment, and 12.8% of participants (14/109) who had been in the program for at least 9 months completed the GAD-7 assessment; 51.5% of participants (68/132) who had been in the program for 6 months were active in the program. Likewise, 43.1% of participants (47/109) who were in the program for at least 9 months remained active. We observed a 3.17-point reduction in GAD-7 scores by program month 3, the end of the intensive phase of the intervention (95% CI –2.13 to –4.21, *P*<.001; Hedges *g*=0.87). Further improvements in anxiety were noted at program month 6 (4.87-point reduction, 95% CI –2.87 to –6.85, *P*<.001; Hedges *g*=1.24) and program month 9 (5.19-point reduction, 95% CI –6.85 to 4.81, *P*<.001).

In the case of both depression and anxiety, participants with higher symptom severity at baseline appeared to show greater postintervention improvement. Higher anxiety scores at baseline predicted greater reduction in GAD-7 scores (β=–0.52, *P*=.001). A similar trend was observed for depression, but the effect did not reach significance (β=–0.47, *P*=.08).

### Engagement

Summary statistics for program engagement during the intensive phase (first 12 weeks) of the program are shown by condition in Table 2. We measured program engagement along two dimensions: extent of contact with the therapist (ie, consultations and number of messages sent by the participant to the therapist) and interactions with other aspects of the digital health intervention (ie, number of completed assessments and number of completed lessons and activities). As expected, we observed a significant positive association between engagement factors (see Table 3). To reduce multicollinearity between factors in the final linear mixed-effects model, each of the engagement variables was first normalized, after which an aggregate engagement score was calculated.

One of the current open questions around digital mental health interventions has been the role of program engagement in facilitating treatment and symptom improvement. We examined if the extent of usage and interaction with the Vida Health cognitive behavior therapy platform was related to mental health outcomes. Indeed, increased program engagement during the intensive phase of the intervention was associated with greater improvement in depression (β=–0.29, *P*=.01) and anxiety (β=–0.29, *P*<.001). Engagement was added in the linear mixed-effects model as a composite factor and was defined a priori, so as to avoid cherry picking factors. In a supplementary analysis, each scaled engagement factor was entered into the model separately. This analysis revealed that therapist consultation, lesson completions, and the number of survey completions were each a significant predictor of PHQ-8 reduction (*P*<.001). However, the number of messages sent to the therapist, while positively associated with the other engagement factors, was not a significant predictor of change in depression and anxiety scores. In other words, activities that did not involve direct interaction with the therapist appear to have also moderated improvement in depression and anxiety. However, as shown in Table 3, we acknowledge that behaviors such as lesson and survey completions may have been facilitated by the therapist, indirectly. Nevertheless, these results suggest a synergistic relationship between app-based features and therapist support.

**Table 2:** Program engagement during the intensive phase of the program by condition.

Engagement factor	Depression (n=139), mean (SD)	Anxiety (n=146), mean (SD)
Survey Completions	5.54 (3.50)	5.75 (3.90)
Therapist Consultations	4.64 (4.86)	4.90 (5.17)
Messages Sent to Therapist	20.66 (22.71)	22.15 (23.43)
Completed Lessons and Activities	19.57 (17.91)	21.88 (19.63)

**Table 3:** Pairwise correlation matrix of program engagement factors by condition.

Condition and factor			Survey completion	Therapist consultations		Messages to therapist	Lessons and activities	*P* value
**Depression**								
	**Survey completion**							.001
		*r*	1		0.32^a^	0.24^a^	0.56^a^	
	**Therapist consultations**							<.001
		*r*	0.32^a^		1	0.63^a^	0.72^a^	
	**Messages to therapist**							.04
		*r*	0.24^a^		0.63^a^	1	0.49^a^	
	**Lessons and activities**							<.001
		*r*	0.56^a^		0.72^a^	0.49^a^	1	
**Anxiety**								
	**Survey completion**							<.001
		*r*	1		0.34^a^	0.28^a^	0.68^a^	
	**Therapist consultations**							<.001
		*r*	0.34^a^		1	0.63^a^	0.69^a^	
	**Messages to therapist**							.006
		*r*	0.28^a^		0.63^a^	1	0.51^a^	
	**Lessons and activities**							<.001
		*r*	0.68^a^		0.69^a^	0.51^a^	1	

^a^The correlation is significant at a level of .05 (two-tailed).

Overall, the extent of engagement (ie, the number of messages sent and lessons completed per week) declined with program time. However, 77.3% (51/66) of participants who completed an assessment at the end of the intensive phase, remained active in the program at month 6 and 58.1% (25/43) were active at program month 9. It was possible that this effect was due to survival bias, in that the program retained participants who were improving. We tested this hypothesis using a logistic regression model to examine if greater reductions in depression at the end of the intensive phase predicted the likelihood of remaining active in the program at month 6. However, changes in depression scores at the end of program month 3 failed to predict the likelihood of remaining active in the program (*P*=.55).

## Discussion

### Principal Results

Vida Health’s digital cognitive behavior therapy program is an app-based structured intervention that includes one-on-one sessions with a remote licensed therapist. The objective of the study was to assess the effectiveness of Vida Health’s digital therapy program in the treatment of mild to moderate depression and anxiety. The analyses revealed significant and clinically meaningful reductions in PHQ-8 and GAD-7 scores relative to baseline, postintervention at 12 weeks, that were then sustained at month 6 of the program. While there is a growing body of evidence that suggests that digital mental health interventions are as efficacious as face-to-face interventions [12], their long-term effectiveness remains an open question. The results of this study suggest that the Vida Health therapy program was associated with improvements in depression and anxiety. Furthermore, the significant association between increased engagement and positive outcomes (depression: *P*=.01; anxiety: *P*<.001) suggests that the interaction with the digital platform beyond just therapist contact was a component in the mechanism of action. However, this study lacked a control or comparison group, and instead, employed an observational study design, thereby preventing the drawing of any causal inferences.

Nevertheless, the results of the present study are consistent with findings from previous research. A meta-analysis evaluating the effectiveness of cognitive behavior therapy-based digital mental health intervention for the treatment of anxiety and depression among young adults reported a mean effect size Hedges *g*=0.72 [25]. Other meta-analytic reviews have observed similarly robust effect sizes for anxiety (Cohen *d*=1.1) and depression (Cohen *d*=0.41) [26]. While there is compelling evidence the digital mental health interventions are feasible, acceptable and effective for depression and anxiety disorders specifically [25,27], it remains unclear if all therapeutic approaches are equally effective. Carolan et al [28] observed that a cognitive behavior therapy–based occupational program was no more effective than interventions that utilized other therapeutic approaches; they hypothesized that cognitive behavior therapy–based digital programs are not designed for delivery in occupational settings. Although the Vida Health cognitive behavior therapy program is not delivered in the workplace, the participants in the study were recruited from their place of employment, and the content of the intervention employed a framework mindful of occupational stress. More specifically, behavioral stress reduction techniques suitable to the workplace such as progressive muscle relaxation and mindfulness practices were integrated into cognitive behavior therapy and delivered as homework via the app.

Previous research also suggests that digital mental health interventions that are self-contained may facilitate improved program outcomes compared to those of hybrid interventions that integrate therapist support and app-based content delivery [8,10]. In this study, we observed strong positive associations between contact with the therapist, defined by consultations or in-app messaging, and overall program engagement, by way of utilization and completion of lessons and activities in the app. While the Vida Health cognitive behavior therapy program can be completed without therapist contact and as a standalone app-experience, we observed that therapist support and comprehensive engagement across the platform was associated with improved outcomes. Overall, the results of this study are encouraging and suggest avenues for improving engagement and achieving enduring positive outcomes.

### Limitations

This study utilized a nonrandomized observational design. As such, this approach precludes any causal conclusions on the effectiveness of the intervention on the observed mental health outcomes. Self-selection bias and the lack of random assignment increase the likelihood of an overestimation of the effect size and limit generalizability of the findings. Furthermore, it was noted earlier that 54.7% of participants (177/323) completed the initial mental health assessment but failed to engage in the intervention, and as a result, these participants were excluded from all subsequent analyses. Attrition is a crucial study limitation and remains a continuing challenge for digital health interventions, particularly those focused on mental health. Fleming et al [29] have noted that uptake and usage of self-guided digital mental health interventions vary significantly, where program completions ranged from 0.5% to 28.6%. While we did not observe significant baseline differences between program nonstarters and participants who continued with the program, it is possible that participants who chose to continue in the program differed systematically from program nonstarters on factors such as the presence of co-occurring health conditions that were not disclosed during onboarding or patient activation and technology acceptance factors that were not assessed during enrollment, thereby limiting the generalizability of the findings.

The Vida Health cognitive behavior therapy program can be completed without interaction with a therapist; however, the program is designed to be therapist supported. It is possible that participants who failed to engage were seeking a self-guided approach. It is also important to note that patients may disengage from the program as symptoms improve; therefore, lack of engagement or incomplete key outcome measures, may not necessarily imply lack of improvement. Another key limitation to the generalizability of this study is the low completion rate of the assessment during the maintenance phase. While participants were encouraged by their therapist and through in-app messaging to complete the assessments, survey completion was mandatory only at the time of program enrollment and was optional at all future time-points. However, it is possible that the semimonthly assessment for the entire study duration prompted survey fatigue. Baseline score severity and changes in depression scores at the end of the intensive phase did not increase the likelihood of assessment completion at program month 6 (*P*=.45) and 9 (*P*=.86). It, therefore, seems unlikely that only participants who improved were motivated to complete future assessments. Furthermore, other tools and activities such as tracking moods, anxiety episodes, and mindfulness minutes were also available throughout the program. It is possible that, in some cases, participants used a multitude of these tools instead of the assessment to track progress. App-based activities in Vida Health therapy program included guided lessons and interactive trackers (eg, tracking mindfulness or meditation minutes). Supplementary analyses revealed that lessons pertaining to maladaptive thinking patterns had the highest rates of completion across participants compared to content related to mindfulness or sleep. It is possible that altering or expanding content to include topics with the greatest uptake may be associated with improved program retention. We also noted that lessons were consistently utilized at a greater rate than tools throughout the study duration. In the intensive phase (ie, 12 weeks), participants utilized 2.64 more lessons than tracking tools. During the maintenance phase of the program, participants completed 3.21 more lessons than tracking tools. While beyond the scope of this paper, additional research is warranted to understand if specific patterns of lesson and tool usage are associated with improved retention, and moreover, if specific guided lessons that incorporate interactive tracking tools or videos are associated with greater program engagement.

The Vida Health therapy program offers a structured approach that combines core concepts from cognitive behavior therapy including cognitive model, guided discovery, behavioral activation, and techniques including mindfulness for addressing maladaptive thinking. While an analysis of which of these features may have contributed to program uptake, engagement, and behavioral outcomes was beyond the scope of this study, future research must be undertaken to better understand how these elements influence program effectiveness. Further exploration into how patterns of program engagement alter as symptoms improve or worsen may broaden our understanding of program uptake and retention from that of binary (engaged or not engaged) to multidimensional understanding. In this study, engagement was defined as the combined additive effect of app-based behaviors and the extent of therapist contact. However, dimensionality reduction techniques such as principal component or factor analysis may provide more nuanced insight into patterns of engagement and possible changes with time.

As health care increasingly leverages technology for delivering remote on-demand care and interventions, research on their effectiveness has largely taken a single condition, single outcome approach. However, mental health conditions such as depression and anxiety are often comorbid with other chronic conditions such as obesity and type 2 diabetes. Approximately 40% of adults are living with multiple chronic conditions in the United States [30]. It is estimated that about one in three adults with type 2 diabetes experience clinically significant symptoms of depression [31,32]. We did not examine possible associations between depression or anxiety and other health factors. However, research suggests the co-occurrence of depression and anxiety with other chronic conditions may negatively impact psychological well-being and disease management. While participants in this study were exclusively enrolled in the Vida Health therapy program, the platform supports integrated care wherein a patient may concurrently seek care from a therapist, while also working on the management of a cardiometabolic health condition. Additional research is warranted in understanding the association between improvements in depression and chronic health outcomes.

### Conclusions

It is estimated that 7.1% of all Americans have experienced at least one major depressive episode, with increased prevalence among women and the highest prevalence observed among young adults (18-25 years) [33]. Similar high prevalence rates have been observed for anxiety [34]. In this study, adults with mild to moderate depression or anxiety were enrolled in a digital cognitive behavior therapy program with remote one-on-one video sessions with a licensed therapist. The results of this study indicate significant and clinically meaningful improvements in depression and anxiety scores relative to baseline that were observed postintervention at 12 weeks and sustained at program month 6. However, participant attrition, the lack of a control group, and observational design were study limitations, and therefore, qualify the generalizability of these findings. Nevertheless, evidence-based digital cognitive behavior therapy interventions such as the Vida Health therapy program show promise in increasing access and providing effective care for the management of depression and anxiety.

## Abbreviations

GAD-7: Generalized Anxiety Disorder, 7-item
HIPAA: Health Insurance Portability and Accountability Act
PHQ-8: Personal Health Questionnaire, 8-item
